# Lack of Dental Education among the People

**Published:** 1900-07-15

**Authors:** R. G. Porter

**Affiliations:** Petoskey


### LACK OF DENTAL EDUCATION AMONG THE PEOPLE.

BY R. G. PORTER, D.D.S., PETOSKEY.

Knowing as we all must the extreme delicacy attached to the handling of this subject, owing to the danger of being criticised, not only by fellow practitioners, but also in danger of being called down by our dental societies for advertising if we are not very careful in the methods employed, you may judge with what feelings of reluctance I consented to write upon this topic.

Nevertheless, this is a subject upon which I am glad to write, since I believe it to be one of the most important of all topics under discussion at the present time.

The object of this paper is not to teach members of the dental profession things they do not know, but rather to call to mind the necessity for more diligent and persistent effort in the discharge of our duty as teachers as well as philanthropists in matters dental.

We believe this subject should receive special attention because all other topics in the general practice of dentistry will more readily take care of themselves, for the reason that they seem more apparently and directly remunerative. In view of the existing conditions as regards the lack of dental knowledge among the people, therefore the paramount importance of this subject as compared with others. This deplorable ignorance of which we are all so familiar, needing only to be mentioned to recall many sad as well as ridiculous demonstrations which come to us daily as practicing dentists.

The masses, with but few exceptions, are ignorant of the value of the organs of mastication in relation to the first great principles of digestion and assimilation; ignorant of the physiological effects of the deciduous teeth in relation to the permanent; ignorant of the bad effects of pathological conditions of the first as well as the permanent teeth and the dire results of such condition upon the health of the individual, be it child or adult; ignorant as to the myriads of germs of various diseases which multiply, both in health and sickness, by geometrical ratio, in what is acknowledged to be the best of all incubators, the mouth; ignorant that an acid reaction in the mouth injures the enamel of the teeth, whether the acid is taken directly into the mouth or is formed there by the decomposition of the food; ignorant that mixing food during mastication and insalivation with the debris common in neglected mouths is very unwholesome; ignorant that cleanliness, *in the mouth*, is next to Godliness, and that broken law or neglected opportunity in dental hygiene is sure to bring a multiplicity of punishments; ignorant that certain simple rules carried out with conscientious regularity will bring a rich reward.

The care of the teeth begins with the cradle. It is deplorable that so many mothers are so ignorant, so careless, so indifferent to the welfare of their children's teeth.

Have we done all that can be done to educate the people? From the dental profession alone must come the so much needed instruction. Every man, woman and child in all this broad land of ours needs a dental education to the extent that they may have an intelligent understanding of the teeth and the importance of taking care of them. The people are crying out to us for more knowledge, and it seems to me that what is needed at the present time is the simplest kind of instruction.

The difficulty seems to be the lack of method or methods to communicate this enlightenment without infringing upon our most valued Code of Ethics. We can not hope *so much* to educate the older men and women on this important subject, but our effort should more especially be spent upon the children, youth and young people.

One plan which seems feasible, and that I would like to present for your consideration, is that this Association appoint a committee whose duty it shall be to see that every school board in all parts of the State be instructed and advised to invite one or more competent dentists from their community to deliver to the public schools during every winter term of school short, boiled down, instructive lectures on dental physiology and hygiene, which shall also be printed in the county papers. This, so far as I have investigated, can be done with but very little expense.

Thus we are not only impressing the teachers with the importance of the question, but educating the rising generation who will soon be parents or teachers, and through the press reach many who are beginning to appreciate their lack of dental knowledge and its importance to them and to their families.

There is also a great opportunity for instruction in our colleges, normal and industrial schools, where hundreds who are fitting themselves for the profession of teaching are going out to take charge of our common schools in all parts of the State.

The publication which is no doubt familiar to the most of you, *Information for Patient and Dentist*, published in Kent, Ohio, is an admirable paper containing many articles each month that are exceedingly useful and right to the point on dental hygiene, written by some of our best men. In the effort to impart instruction through the teachers to the scholars of the common schools, we found the teachers, without an exception, willing and anxious to communicate this knowledge to their several classes the results of which, in numerous cases brought to our attention, have been very gratifying.

We believe also in every dentist having cards or small folders upon which may be printed for instance: No. 1. Instructions to the mother on the care of the teeth of children. No. 2. Simple, brief instruction on the care of natural teeth for adults. No. 3. Reasons for proper care of artificial dentures.

These will be found of benefit to the patient and save the dentist many words and much time. Can we expect the people to be protected from the wiles of the dental charlatan until they are sufficiently educated to intelligently discriminate between intelligent and skillful effort, and the bungling snob who is practicing a profession for revenue only?

[Dr. Porter read from printed slips three different statements he had prepared for distribution to his patients.—REPORTER.]

NO. 1—TO MOTHERS.

Remember, that you are *responsible* for the condition of your child's teeth.

By *nature* a child's teeth should not decay, and should in all cases be kept free from decay. They should be retained and kept sound.

First. Because the jaw is not retarded in its natural growth, and space is made for the second or permanent teeth, which are consequently not so liable to come in crowded or irregular.

Second. If the teeth decay it will cause discomfort in chewing, and the natural tendency will be to swallow without mastication. This will injure the child's digestion and endanger his health.

Third. Any decomposing substance in the mouth, either from decay of the teeth or of food stuffs having been left from a previous meal, mixed with food and swallowed by the child, is detrimental and may bring about some form of disease.

The first or temporary teeth shou'd number twenty.

The first permanent molar tooth the child gets when about six years old—a large tooth which comes just back of the temporary molar teeth, *before* they are shed (or come out). This tooth is *permanent* and is never replaced by another, and is also quite liable to decay. *Look* for it and look to its needs.

See that the child's mouth is cleansed after each meal with a soft cloth, when very young, and with a small brush and water after teeth erupt.

Take the child to your dentist from two to four times a year for examination.

Teach the child when old enough to take care of his *own* teeth.

NO. 2—IMPORTANT.

First. Remember that your health depends largely upon the condition of your teeth.

Second. That mastication is the first and a very important principal in digestion.

Third. That germs of disease of all kinds form rapidly in the mouth and may be the cause of sickness.

The following simple directions are to assist you in the preservation of your health and natural teeth, which, if followed with persistent regularity, would prevent decay of the teeth:

Cleansing the mouth, using a quill tooth pick or floss silk and a good brush after each meal.

If necessary, and prescribed by your dentist, use a tooth powder or a mouth wash.

Have your teeth examined at least twice a year.

---

The President called upon Dr. Hoff to open the discussion.

Dr. Hoff: I fear our President is not only short in stature but also short-sighted in asking me to open the discussion of this paper, as he well knows my mind is not training in this direction exactly, my business is teaching students to be useful as professional men or practitioners, and not that of educating the public. And so if I seem a little off and drop into my familiar theme in considering this subject, I trust you will bear with me.

I do not know that I have anything to say that is specially important on this subject. It is an important subject, however, and any one who is interested in his profession and the practice of same, or one who is interested in the welfare of the public in dental hygiene, it is an exceedingly interesting topic and one that all ought to have something to say about.

That the public needs education in this direction there is no question in my mind, and there should be none in the mind of any one who has practiced dentistry for any considerable time. I do not care how intelligent a community is, we are continually being brought face to face with what seems to us gross ignorance on matters of oral hygiene, with which it seems every one ought to be entirely familiar. The ordinary prophylactic measures that every one is supposed to make use of in making an ordinary toilet are oftentimes grossly neglected, or, if not neglected, are accomplished in such a way as to be worse than useless in their results. Take the simple brushing of the teeth, how many of our patients really know how to brush the teeth to make them clean? Many dentists, who are intelligent, and who ought to be well informed in reference to the skillful selection of a tooth brush, never seem to think their patients need instruction.

The matter of tooth powder and tooth wash which are intended to cleanse the teeth is another matter that needs a great deal of attention. We have myriads of tooth powders and it is sometimes puzzling when patients ask, "What powder shall I use, or what do you recommend?" If I should ask the question here to-day, I might receive as many answers as there are people in the room. These things are essential, the method of using the tooth brush and application of tooth powder; they are of the utmost importance in the preservation of the teeth. I have no doubt that all of you have seen cases where the gums had been grossly abused by the careless use of the tooth brush, or by the use of excessively gritty tooth powders; the gums have been made to receed from excessive manipulation. The reasons for such injuries and the way to prevent them is a matter which not only the profession but the public needs to know, particularly the young who are just at that period in their lives when their teeth need a great deal of such attention.

While I will not take your time to give my ideas of the proper methods of selecting tooth brushes or tooth powders, it seems to me along this line the public needs a great deal of education.

More important, however, it seems to me is the education of the public as to conditions which arise later in life. If we would take the disease which probably puzzles and tries our patience more than any other, Pyorrhea Alveolaris. Some one has said this is a filth disease, but it is not always so, it does not always come as a result of neglect of the mouth, but if we are to have success in the restoration to normal condition of mouths of this kind, it will be necessary to give close attention to hygienic treatment. We may use our utmost therapeutic and operative skill to restore the tissues to their normal condition, but if we neglect the important matter of cleanliness in the mouth our work will be fruitless, no permanent results, not even temporary results are assured. Our patients must understand this, we need their co-operation to secure any sort of adequate results in the treatment of this condition.

Again, in the treatment of those cases where, because of the loss of occluding teeth, the articulation of teeth is at fault, because they have readjusted themselves into abnormal positions in the arch, or to those cases in which there is a lack of restoration of contour in fillings from bad manipulative procedures; the patient's attention should be directed to these conditions and taught to care for them.

When patients ask for a phosphate filling, because they can not afford some other, it is the business of the dentist to persuade such patients that a filling of that sort, while it may be inexpensive is really unsuitable, not merely because you can make a larger fee from another operation that you advise it, but that the case needs just that kind of an operation for the good of the mouth.

The education of patients in this direction may or may not be profitable financially, it will be for the betterment of our patients' mouths, and patients as a result of this advice will appreciate the services which we have rendered so much that they will be perfectly willing to pay fees for such advice and services, and be so grateful for it that they will be willing to place themselves entirely in your hands for further treatment.

This matter of whether we are going to render ourselves liable to be held up for infringement of the Code of Ethics, or make ourselves obnoxious to our fellow-practitioners is a kind of bugbear which no honest minded man should be afraid of. Every practitioner should do some such work as Dr. Porter has outlined, it is admissible from the professional standpoint, it is professional, it is the highest order of professional work. I can readily see how an unscrupulous man can practice such a thing and make it an advertising scheme. But no honest man who loves his patients and his work will ever make such a mistake as that. If I interpret Dr. Porter's idea properly, and I think I do, he is doing this not for his own sake, not that he can build up a little more practice for himself and other dentists in his community, but it is purely a humanitarian idea and one that deserves the highest order of commendation, and as such I stand here to-day to encourage him in it. I have never done anything of this sort and am rather ashamed of it; I talk to my patients, but never have done so in print.

Dr. Hall: I was very much taken with the point Dr. Porter brought out in reference to what might be done in the schools.

Those who have little children have no doubt noticed how they talk about what they have learned in the schools. It is wonderful the impression the teachings of the present text-books make upon children's minds, particularly the text-books of physiology, which warn them of the dire effects of using tobacco, drinking tea and coffee, etc. I do not myself believe in that sort of teaching, yet there is no doubt that the child remembers the facts. I refer to this to illustrate how things put in this way attract children's attention, and if such a paper as Dr. Porter refers to could be sent to the schools and the teachers would read extracts from it I think it would be very useful; indeed, it would set them to thinking at least.

There should really be an examiner to go to the different schools and examine each child's mouth. There are plenty of people who can afford it and do send their children to the dentist to have their mouths looked after; on the other hand, there are many who do not have the money and consequently do not send their children to the dentist. I believe the local societies and associations could do a great deal in the way of advising school boards to have some sort of board of examiners to look after the children's teeth.

DR. ROAT: The point of educating the children in this line in the public schools is a very interesting one to me. I have a boy six years old, he goes to school, and every day when he comes home he has something very interesting to speak about, and if it was anything to do he would turn the house upside down in order to do it. He is bound to carry that instruction out. For that reason the children can be better taught in school and instruction could be given them which could not be done at home. The average parent could talk himself blind, so to speak, and not impress the child as would one word from the teacher. For that reason I heartily favor this idea of instruction on dental subjects in the schools.

DR. METCALF: Unfortunately for me I was not present to hear the whole of this paper by the gentleman, but in looking over one of the slips headed "Important" I find that the most important thing that should be impressed upon parents is not here in this paper at all, and that is this one fact—that whenever you keep the teeth clean, absolutely clean, they can not decay—that is the thing that should be impressed upon the minds of the parents and children.

DR. CROUSE: It strikes me that the most important thought in connection with this subject has not been touched upon. We know that teeth decay at one time of life and are immune at another. We know that in the same family the teeth of one child will decay more than those of another, with the same kind of food. We know that the most delicate looking individuals will have the least difficulty with caries of their teeth, and *vice versa.* Now, what is the great problem which the dentist has to solve to-day? It is this: Why are teeth immune from decay under so many different circumstances? Miller and others have discovered that the decomposition of starchy matter is brought about by micro-organisms, which produces an acid and causes cavities to form in the teeth. Now, we have the starchy matter in some mouths, and we have the microbes and not any cavities. I have in mind a young lady who has been in my care for twenty years. She came to me when a baby, she was very delicate, had curvature of the spine, wore a brace for a time, yet she never had a symptom of decay in her teeth. In the same family is a young man who is very vigorous, yet his teeth are never immune from decay, it is going on all the time. Now, why this difference? When we get to the point where we know what brings about this difference we will be able to state some facts, and something will be accomplished in the way of overcoming decay.

When I first commenced practicing in Chicago there was a journal published by the dentists to educate the community, but it soon aroused jealousy and a great fight; the dentists who were not in it thought the others were trying to take advantage of them. I was too young to appreciate the quarrel, but the journal wound up in defeat.

I think a proper text-book in reference to this would be a good thing. I must say I have been struck with the idea of preparing a book on "How to Conduct a Practice," which would involve this very question of how to educate your patients. It would be one of the most useful things, but I do not see my way clear yet for lack of knowledge.

I think the best place to get at your patient is at your chair; get hold of the mothers and get them interested enough to appreciate their own teeth, and now we come to an important subject and one which is very little understood.

I venture if I examined the mouths of the people in this house I would not find two clean. How many patients come to your office with their teeth clean? It is a rare thing that the teeth are brushed as they ought to be. I am in the habit of getting a mouth mirror and hand glass—it is the first thing I do—and I show them, so they do not dare to come again to my chair until they have brushed their teeth.

I saturate a pledget of cotton with a little peroxide and apply it to teeth and show it to patients, the next time they come their teeth will be better brushed, and the same is true with children. I have been practicing in Chicago thirty years, I have mothers, children and grandchildren, and it has been my pride to now and then get a family to come to me with their teeth brushed; when I have accomplished that I think I have done about as much as any one thing I can do. When a child gets into the chair and is sent home once or twice and the importance of brushing his teeth is impressed upon him, he returns with clean teeth. I often send them home with a note which reads, "I have sent this child home to have his teeth brushed, he need not return until he does."

VOICE: How much do you charge for that appointment?

In doing this I think I am doing just as much service as a physician who writes a prescription or a lawyer who give a little advice and charges a handsome fee.

I make the thought of unclean teeth just as disgusting as I can, and I come very near doing it. That will go a great deal further and the sister and brother of that child will get onto the racket, and they will take the chair with a great deal more fear on that subject.

So I say the most important thing for us is to find out what it is that causes this condition of caries in the mouth. Once in a while we get hold of a child without any caries; we do in the city, and I presume it is more so in the country. It is something we do not understand, why should it take place in the mouth at one time of life and not at another, and *vice versa*.

Dr. Porter: I thank the gentlemen very much for their criticisms, they are to the point. Although there are a great many things that we are not acquainted with, we should not wait for this reason until we make more investigations, but should teach our patients facts which we know at the present time.

I find that I have not the time to explain these facts to the patients at the chair, neither is their brain in a fit condition to receive same, but on taking them home they can read at their leisure.

These slips were gotten up in a great hurry, and I must apoligize for such an expression as criticised by Dr. Metcalf. But they do good and are appreciated more than a thousand words at the chair.

Adjourned to 7:30 P. M.

Monday evening's session met at 7:30 P. M.

Minutes of previous session were read and adopted as corrected.

The Treasurer then called roll, and the Board of Censors reported long list of eligible candidates for membership. On motion the rules were suspended and all were elected by unanimous vote.

A recess of ten minutes was ordered by the President for payment of dues and reception of new members.

On reassembling, the Association by vote made Wednesday at 11 o'clock a special order of business to visit the Michigan Insane Asylum in a body.

Dr. Hoff then read the following paper on "Gold as a Filling Material."

